# How averse are the UK general public to inequalities in health between socioeconomic groups? A systematic review

**DOI:** 10.1007/s10198-019-01126-2

**Published:** 2019-10-24

**Authors:** Simon McNamara, John Holmes, Abigail K. Stevely, Aki Tsuchiya

**Affiliations:** 1 School of Health and Related Research (ScHARR), University of Sheffield, Sheffield, S1 4DA UK; 2 Department of Economics, University of Sheffield, Sheffield, S1 4DA UK

**Keywords:** Health inequality aversion, Social preferences, Equity weighting, Fair innings, Systematic review, I14, D04

## Abstract

There is growing interest in the use of “distributionally-sensitive” forms of economic evaluation that capture both the impact of an intervention upon average population health and the distribution of that health amongst the population. This review aims to inform the conduct of distributionally sensitive evaluations in the UK by answering three questions: (1) How averse are the UK public towards inequalities in lifetime health between socioeconomic groups? (2) Does this aversion differ depending upon the type of health under consideration? (3) Are the UK public as averse to inequalities in health between socioeconomic groups as they are to inequalities in health between neutrally framed groups? EMBASE, MEDLINE, EconLit, and SSCI were searched for stated preference studies relevant to these questions in October 2017. Of the 2155 potentially relevant papers identified, 15 met the predefined hierarchical eligibility criteria. Seven elicited aversion to inequalities in health between socioeconomic groups, and eight elicited aversion between neutrally labelled groups. We find general, although not universal, evidence for aversion to inequalities in lifetime health between socioeconomic groups, albeit with significant variation in the strength of that preference across studies. Second, limited evidence regarding the impact of the type of health upon aversion. Third, some evidence that the UK public are more averse to inequalities in lifetime health when those inequalities are presented in the context of socioeconomic inequality than when presented in isolation.

**Electronic supplementary material:**

The online version of this article (10.1007/s10198-019-01126-2) contains supplementary material, which is available to authorized users.

## Introduction

The UK is an unequal society. If you are poor, you can expect to live a shorter life than if you were rich [1, 2], you can expect to live with lower average health-related quality of life [3], and you can expect to experience disability at a younger age [4]. This “health gap” is substantial [5]. In quality-adjusted life-year (QALY) terms, a person living in the most deprived quintile areas of English society can expect to experience 11.87 QALYs less in their lifetime than a person living in the least deprived areas [3].

Recent evidence suggests the UK public are averse to this inequality, and would be willing to sacrifice a significant amount of average population lifetime health to achieve a more even distribution of it between socioeconomic groups^1^ [6–9]—they appear to be “distributionally sensitive”. In contrast, economic evaluation in health is typically “distributionally naïve” [10], and operates under the assumption that “a QALY is a QALY is a QALY” [11], irrespective of who receives it. This apparent discordance has led some to question the democratic legitimacy of distributionally naïve approaches, and to call for distributionally sensitive forms of economic evaluation, such as “distributional cost-effectiveness analysis” [12–14].

If the UK public’s preferences towards inequalities in health are to be captured in distributionally sensitive economic evaluation, it would be valuable to understand the answers to three questions: (1) How averse are the UK public towards inequalities in lifetime health between socioeconomic groups? (2) Does the extent of that aversion differ depending upon the type of health (e.g. life extension, pain relief or mobility improvement) under consideration? (3) Are the UK public as averse to inequalities in health between socioeconomic groups as they are to inequalities in health between neutrally framed groups? This third question matters, as it is not immediately obvious whether or not it is normatively desirable for social health-related resource allocation decisions to be made based on socioeconomic status, or whether they should be based on health alone [15]. This systematic review focuses on these three questions.

Previous systematic reviews have focused on general public preferences regarding different broad criteria for prioritisation [16, 17], or preferences regarding differences in the future health of individuals [18–20]. This is the first systematic review to focus explicitly on the UK public’s aversion to inequalities in lifetime health between socioeconomic groups, although an unsystematic review has recently been published [21]. The scope of this review is restricted to the preferences of the public in the UK, as the primary objective of the study is to inform distributionally sensitive economic evaluation in the UK.

## Methods

### Search strategy

Four databases were searched: Ovid MEDLINE (1946—27/10/2017), Ovid EMBASE (1974—26/10/2017), Ovid EconLit (1886—30/09/2017), and Web of Science’s Social Sciences Citation Index (SSCI) (1956—27/10/2017). All searches were undertaken on 27/10/2017.

The search strategy was developed in an iterative fashion. First, six “pearls” [22] were identified as starting points, to provide the initial list of key words [6, 8, 23–26]. Second, the MeSH headings associated with these papers were recorded, and a word frequency analysis of the paper titles/abstracts was undertaken [27]. These were supplemented with additional terms based upon the search questions to generate an initial search strategy.^2^ Following this, the reference lists of the pearls were reviewed, to identify additional papers. The sensitivity of the draft search strategy was then tested in MEDLINE, by assessing whether or not it could return the papers identified from those reference lists. If a paper was not identified, the search strategy was then updated with key terms from the unidentified paper. Further scoping searches were then conducted based upon this revised strategy, and the reference lists of potentially relevant papers scanned for other potentially relevant papers.

The search strategy was then tested again to assess whether it identified all papers identified in scoping searches, refined as needed, and the same process repeated until the reference list of all papers identified in scoping searches were picked up by the search strategy. The final MEDLINE search strategy is detailed in Online Appendix 1. Following the screening of the database search results, the selected papers were reviewed in detail, to identify potentially relevant journal publications, or grey literature, not captured within this search. These papers were then treated as new records, and screened accordingly.

### Eligibility criteria

Papers were assessed for eligibility using six hierarchical inclusion criteria. First, papers published in English were included, and all others were excluded. Second, publications in peer-reviewed journals, reports published by NGOs/HTA bodies, and studies published in discussion papers by academic institutions, were included. All other publication types, including conference abstracts, were excluded. Third, experimental studies in which the stated preferences of participants were quantitatively elicited were included. Non-experimental revealed preference studies, non-quantitative studies, and reviews of prior studies, were excluded. Fourth, those studies featuring broadly representative samples of the UK adult general public were included.^3^ Studies centred on selective samples of the UK population, such as students, policy makers and health care professionals were excluded. Studies featuring exclusively non-UK respondents, or for which it was not possible to isolate the preferences of UK respondents, were excluded. Fifth, studies were assessed for their ability to provide information on the extent of the public’s aversion to inequalities in lifetime health between socioeconomic groups. Studies that explicitly asked, or could be implied as asking, respondents to make efficiency/equality trade-offs between individuals, or groups, with differing lifetime health in a range relevant to socioeconomic inequalities in health (life expectancy^4^ or quality-adjusted life expectancy: > 50 and < 90 [1]) were included—irrespective of whether participants were told they were choosing between socioeconomic groups, or between neutrally framed groups in a comparable range of lifetime health. Two distinct strands of empirical literature were considered to be capable of providing this information—(1) stated preference studies focused on health inequality aversion^5^ [28–30], and (2) stated preference studies focused on eliciting preferences regarding prioritising those individuals with a higher Burden of Illness, as defined by their absolute QALY shortfall in prospective health attributable to some illness^6^ [31, 32].

Studies that did not apply a lifetime time-horizon, or that could not be utilised to infer aversion to lifetime health, were excluded. Stated preference studies that focused on severity, as defined by relatively poor quality of life [19], and preferences regarding treatment at the end of life [18] were excluded for this reason. Studies focused explicitly on inequality aversion in the context of gender, or differences in lifestyle, were also excluded. Finally, the choice perspective employed in each study was evaluated. Those studies that asked respondents to make choices in the context of public resource allocation decisions that did not affect them personally, for example how to allocate finite NHS resources between two groups they were not part of, were included. Those studies that asked respondents to make choices that would impact them, for example their willingness to trade-away their own wealth, were excluded.

### Study selection

Study selection was conducted using a two-step process, with titles and abstracts screened first followed by screening of full papers. Eligibility criteria were applied sequentially in the order detailed above, with the first arising reason for exclusion recorded. The first two waves of screening were conducted by Simon McNamara. Abigail Steveley then independently reviewed a random sample of 20 full papers against the eligibility criteria. This independent review identified one discrepancy: the decision of whether or not to include a study by Petrou et al. [33]. The lead author of the study was contacted to clarify whether it used a general population sample, which resolved the discrepancy and the study was included. The audit identified no significant concerns regarding the screening undertaken.

## Results

### Search output

**Fig. 1:**
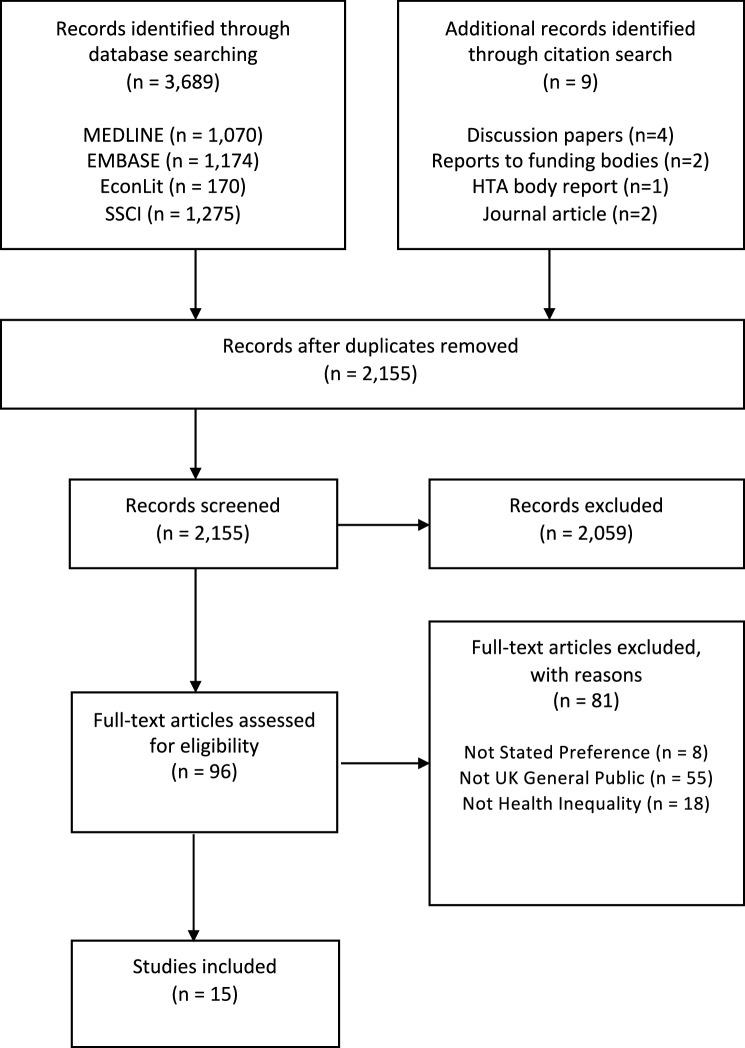
PRISMA Flow Diagram

In total, 2155 unique records were screened after removing duplicates. Of these, 2059 were excluded based upon title and abstract alone, and 96 full-text articles were retrieved. Of these, 81 were excluded, leaving 15 final records (Fig. 1) [34]. The commonest reason for exclusion of full-text articles was the study population. Most of these excluded records were based on studies conducted in other countries, although a proportion were conducted in selective samples of the UK population, such as students or healthcare professionals. The conduct of the search, and rationale for exclusion of papers, is detailed in a PRISMA flow-chart, above [34].

### Characteristics of included studies

#### Choice context

Of the 15 studies identified, 8 provided estimates of aversion to inequalities in health between neutrally labelled groups [23, 24, 31, 33, 35–38], whilst 7 provided estimates of aversion to inequalities in health between socioeconomic groups [6–9, 39–41].

#### Participants

Forty percent of identified studies recruited local samples, whilst 66.6%^7^ recruited national samples. The identified studies ranged in size from only 26 participants [40], to 3669 participants [31]. On average, those studies that provided estimates of inequality aversion between neutrally labelled groups were substantially larger than those that provided evidence of aversion between socioeconomic groups (mean *n* = 1064 vs. *n* = 154).

#### Mode of administration

The studies used a wide range of administration modes. These included individual interviews—both computer assisted [35, 36],^8^ and paper-based [8, 24, 38]—postal questionnaires [9, 39], online studies [6, 7, 33, 37], and discussion groups featuring individual completion of choice exercises [7, 23, 40]. Of the discussion groups, one was a NICE Citizen’s Council [40].

#### Methods

The 15 studies applied a variety of different methods. Four studies utilised a form of benefit trade-off (BTO), based upon a design first developed by Shaw et al. [6–9, 28]. Two studies applied discrete choice experiments (DCEs) [36, 37] featuring multiple attributes, two featured a person trade-off (PTO) exercise [33, 35], and two featured simple choice questions^9^ [39, 40]. Three studies used a form ranking exercise [33, 38, 41], whilst the remaining two studies featured other forms of choice exercise [24, 37].

Whilst a range of different methods were identified, these were not spread evenly across choice contexts. Both the DCEs [36, 37], and the two PTO [33, 35], studies elicited only aversion to inequalities in lifetime health between neutrally labelled groups, whilst all of the four Shaw et al. variant BTO studies [28] elicited aversion between socioeconomic groups.

The identified studies explored a range of different measures of lifetime health, including life-expectancy at birth [8, 9, 39, 41], age at death [33, 38], expected number of lifetime QALYs—presented as either decomposed profiles^10^ [23, 35, 36], or composed values [24], BOI as expressed by the QALY [31, 37]—and the number of “years of life in full health over the average person’s lifetime” [6, 7]. In those studies that elicited aversion to health inequality between socioeconomic groups, the labels given to the groups included: “the richest” and “the poorest” fifth of society [6, 7]; having a “wealthy background” or a “poor background” [39]; and social (occupational) “Class I” vs “Class V”^11^ [8, 9, 41] (Tables 1, 2).

**Table 1:** Identified studies—study characteristics

Authors (date)	Sample size	Sample population	Administration method	Method	Focus of relevant questions within study
Ali et al. (2017) [7]	135	York + UK	Group with individual response + online	BTO	Aversion to inequalities in YFH
Anand and Wailoo (2000) [39]	144	Leicester	Postal	Simple choice	Relevance of cause of inequality
Baker et al. (2010) [35]	587	England	CAPI	PTO	Social value of the QALY
Dolan and Tsuchiya (2005) [23]	100	Sheffield	Group with individual response	Ranking	Relevance of past/future health
Dolan and Tsuchiya (2011) [8]	130	York	Interview	BTO	Aversion to inequalities in LE
Edlin et al. (2012) [24]	559	England + Wales	Interview	Other choice	Relevance of cause of inequality
Lancsar et al. (2011) [36]	587	England	CAPI	DCE	Social value of the QALY
NICE (2006) [40]	26	England + Wales	Group with individual response + Citizens Council	Simple choice	Prioritising the socially disadvantaged
Petrou et al. (2013) [33]	2500	UK	Online	PTO + ranking	Fair innings
Rowen et al. (2016) [37]	371	UK	Interview + online	Other choice	BOI
Rowen et al. (2016) [31]	3669	UK	Online	DCE	BOI
Robson et al. (2017) [6]	244	England	Online	BTO	Aversion to inequalities in YFH
Tsuchiya et al. (2003) [38]	140	York	Interview	Ranking	Fair innings
Tsuchiya and Dolan (2007) [9]	271	UK	Postal	BTO	Aversion to inequalities in LE
Tsuchiya and Dolan (2009) [41]	128	Sheffield	Group with individual response	Ranking	Aversion to inequalities in LE

*BTO* benefit trade-off, *PTO* person trade-off, *DCE* discrete choice experiment, *CAPI* computer-assisted personal interview, *LE* life expectancy at birth, *YFH* years of life in full health over the average person’s lifetime, *BOI* burden of illness

**Table 2:** Identified studies—context

Authors (date)	Tested inequality	Range of relevant inequality	Tested change
Ali et al. (2017) [7]	YFH	YFH: 62–74	YFH
Anand and Wailoo (2000) [39]	LE	LE: 70–84	Priority
Baker et al. (2010) [35]	LT QALYs (DC)	LT QALYs: < 76	LT QALYs (DC)
Dolan and Tsuchiya (2005) [23]	LT QALYs (DC)	LT QALYs: < 66	LT QALYs (DC)
Dolan and Tsuchiya (2011)^b^ [8]	LE	LE: 73–78	LE
Edlin et al. (2012) [24]	LT QALYs (C + DC)	LT QALYs: 52–76	LT QALYs (C + DC)^a^
Lancsar et al. (2011) [36]	LT QALYs (DC)	LT QALYs: 60–80	LT QALYs (DC)
NICE (2006) [40]	General health	–	Priority
Petrou et al. (2013) [33]	Age at death	Age at death: 60–90	Extra years at full health
Rowen et al. (2016)^c^ [37]	BOI QALYs (DC)	Absolute QALY burden framed	QALYs (DC)
Rowen et al. (2016)^c^ [31]	BOI QALYs (DC)	Absolute QALY burden framed	QALYs (DC)
Robson et al. (2017) [6]	YFH	YFH: 62–74	YFH
Tsuchiya et al. (2003) [38]	Age at death	Age: 55–70	Age at death
Tsuchiya and Dolan (2007) [9]	LE	LE: 73 vs 78	LE
Tsuchiya and Dolan (2009) [41]	LE	LE: 73 vs 78	LE

*BOI* burden of illness, *LT* lifetime, *YFH* years of life in full health over the average person’s lifetime, *DC* decomposed (QALY profile presented in terms of LE, and QoL, but not as a unified figure), *C* composed (QALY figure presented)

^a^[24] involves choices between different profiles, not changes in existing profiles, so this is technically not a “tested change”

^b^Note that [8] also tested aversion between the “healthiest” and “unhealthiest” quintiles of society, these labels are ambiguous and may be interpreted as reflecting the lifestyle of these groups, their lifestyle and their outcomes, or their outcomes alone. As a result, they were excluded

^c^Note that both Rowen et al. papers take a forward looking, rather than lifetime perspective—these studies are included under the assumption that BOI has a linear impact upon the preferences of the public (see “Eligibility criteria” for further detail)

### Findings of identified studies

Of the 15 identified studies, 8 provide evidence of health inequality aversion [6–9, 24, 33, 38], 2 provide evidence of no aversion [36, 39], and 5 provide mixed evidence [23, 31, 35, 37, 40]; see Table 3 above. Seven studies explored aversion between socioeconomic groups, and eight explored aversion between neutrally framed groups.

**Table 3:** Identified studies—summary of results

Authors (date)	Choice context	Evidence of aversion to inequalities in lifetime health?	Atkinson (*ɛ*) parameter^a^ [42]	Weight placed on a marginal gain to group with lower lifetime health^e^
Ali et al. (2017) [7]	Socioeconomic groups	Yes	10.87 or greater	6.8–∞
Anand and Wailoo (2000) [39]	Socioeconomic groups	No	1 (implied)	1
Baker et al. (2010) [35]	Neutrally framed groups	Mixed	–	–
Dolan and Tsuchiya (2005) [23]	Neutrally framed groups	Mixed	–	–
Dolan and Tsuchiya (2011) [8]	Socioeconomic groups	Yes	28.9	166.22
Edlin et al. (2012) [24]	Neutrally framed groups	Yes	5.76–7.63	2.77–3.86
Lancsar et al. (2011) [36]	Neutrally framed groups	No	1 (implied)	1
NICE (2006) [40]	Socioeconomic groups	Mixed	–	–
Petrou et al. (2013) [33]	Neutrally framed groups	Yes	> 1 (implied)	> 1
Rowen et al. (2016) [37]	Neutrally framed groups	Mixed	–	–
Rowen et al. (2016) [31]	Neutrally framed groups	Mixed	–	–
Robson et al. (2017) [6]	Socioeconomic groups	Yes	10.95	6.95
Tsuchiya et al. (2003) [38]	Neutrally framed groups	Yes	> 1 (implied)	> 1 (implied)
Tsuchiya and Dolan (2007) [9]	Socioeconomic groups	Yes	> 1 (implied)	> 1 (implied)
Tsuchiya and Dolan (2009) [41]	Socioeconomic groups	Yes	> 1 (implied)	> 1 (implied)

^a^Atkinson inequality aversion parameters are sometimes presented as “*r*” values, and sometimes presented as “*ɛ*” values. *ɛ* = *r* + 1

^b^Estimates derived based upon baseline inequality tested in [7] and [8]; 62 YFH vs 74 YFH. Atkinson inequality aversion parameters applied where possible—see [8]

#### Aversion to inequalities in health between socioeconomic groups

The seven studies that explored aversion between socioeconomic groups provide general, although not universal, evidence of aversion to inequalities in lifetime health across socioeconomic groups. Five provide support for inequality aversion [6–9, 41], one study provides mixed evidence [40], and one was opposed [39]. In those studies that provide evidence of aversion between socioeconomic groups, the strength of this preference was high. For example, Dolan and Tsuchiya [8] find that participants valued a marginal life-expectancy gain provided to an individual with a social class V (unskilled) occupation and a life-expectancy of 73, between 6.8 and 9.94 times that of a marginal gain provided to an individual with a social class I (professional) occupation with a life-expectancy at birth of 78. Ali et al. [7] estimate relative weights of 6.8 to ∞^12^ on marginal gains, in response to questions asking respondents to allocate incremental gains in “years in full health over the average person’s life” (YFH), to a poor individual with a YFH of 62 years, compared to a rich individual with a YFH of 74 years. For the same comparison, Robson et al. [6] find relative weights of 6.20–6.95.^13^ In contrast, studies where aversion between socioeconomic groups was elicited using alternative methods found more mixed results. Anand and Wailoo [39] find only 8% of respondents felt that a poor individual, who has a life-expectancy of 70 years, should receive priority for the treatment of a disease over a rich individual, who has a life-expectancy of 85 years. The overwhelming majority (92%) believed the two should be treated equally.

One study—a NICE Citizens Council report—provided mixed evidence of aversion between socioeconomic groups [40]. In this study, a minority (40%) of respondents agreed that NICE should “issue guidance that concentrates resources on improving the health of the whole population … even if there is a risk of widening the gap between socioeconomic groups”, whilst a majority (60%) were in favour of focusing resources on “the most disadvantaged members of our society” (p. 15). However, in the same study, 83% of participants agreed with the, seemingly contradictory, statement that “NICE should issue guidance that concentrates resources on where it will have the greatest impact on the whole population” (p. 23), and only 50% agreed with the statement “It is the responsibility of the NHS to attempt to narrow the gap between the least and most disadvantaged in our society in terms of public health” (p. 24).

#### Aversion to inequalities in health between neutrally labelled groups

Eight studies explored aversion to inequalities in lifetime health between neutrally labelled groups, in a range of lifetime health comparable to those tested in socioeconomic group framed studies (a quality-adjusted life expectancy or life expectancy > 50 and < 90 [1]) [23, 24, 31, 33, 35–38]. Three of the eight studies provided support for inequality aversion [24, 31, 33, 38], albeit at lower levels than identified in those studies focused on aversion between socioeconomic groups.

Edlin et al. [24] provide the highest estimate of aversion to inequalities in health between neutrally labelled groups. In this study, the authors tested aversion to two inequalities. In the first of these, “study state A” (68 QALYs vs 54 QALYs) the authors found respondents granted a weight of 3.1 to an incremental health gain to the worse off group. In the second, “study state B” (76 QALYs vs 52 QALYs) the authors found a weight of 3.5.^14^ In contrast, Petrou et al. [33] estimate a weight of only 1.37,^15^ on a 5-year life extension at perfect health, provided to someone who would otherwise die at age 60 years, compared to someone who would otherwise die at age 80 years. This finding is consistent with that of Tsuchiya et al. [38], who found the public were willing to prioritise granting a 5-year survival benefit to a 55-year old who will otherwise die immediately, over an equivalent gain to a 70-year old, albeit without estimating a precise weight on the strength of that preference.

Four of the eight studies provided mixed evidence of aversion to inequalities in health between neutrally labelled groups. Two of these [31, 37], were focused on quantifying public preferences towards granting priority to those individuals who have a higher burden of illness (BOI), as expressed by their QALY shortfall, over those with lower BOI. In the smallest of these two studies [37], the authors asked four questions relevant to this topic.^16^ Three of these questions provided no support for granting preference to those who had a higher BOI, whilst one provided modest evidence (59% support) of a preference towards prioritising the worse off. In the largest study [31], the same research team found evidence of a preference towards treating those with higher BOI, over those with lower BOI—implying an aversion to inequalities in lifetime health. However, when they then deconstructed the impact of BOI into that attributable to loss of life-expectancy, and loss of health-related quality of life, the authors found respondents preferred to prioritise those whose BOI was attributable to loss of length of life, and made the opposite choices about those who BOI was due to losses of health-related quality of life^17^—a finding consistent with the fair innings hypothesis, but not the extended fair innings hypothesis [30]. This finding is similar to that observed by Dolan and Tsuchiya [23], who found preferences consistent with aversion to differences in life-expectancy, but not quality-adjusted life-expectancy. In both of the questions that Dolan and Tsuchiya tested, participants ranked the opportunity to provide a health benefit to the individual with the lowest lifetime QALYs second to last out of the six options tested. This outcome appears to have primarily been driven by the fact that respondents were not as averse to differences in past-quality of life as would be suggested by the QALY model, and placed a much higher emphasis on length of life, than lifetime quality of life.

Baker et al. [35] also find mixed evidence on inequality aversion. In their PTO study, the authors evaluated respondents’ preferences towards granting an incremental health gain to individuals who are expected to die at differing ages. This gain took the form of a 20% gain in health-related quality of life for their last 20 years of life (4 QALYs). In response to these questions, the authors found respondents preferred to give the incremental benefit to individuals who are due to die at age 60 years, rather than those who are due to die at age 80 years—with an estimated relative weight of 1.55^18^ on the gain provided to those with lower lifetime health. However, in the same study, the authors conducted a series of “profile tests” in which the lifetime health of certain profiles was varied to test the extended fair-innings hypothesis (e.g. by changing past quality of life, or by granting the profiles additional length of life after the tested quality of life gain). In these profile tests, the authors find mixed results, with, if anything, “a tendency to favour those with higher lifetime health” (p. 45).

The sole study to provide evidence of no aversion to inequalities in health between neutrally labelled groups was that by Lancsar et al. [36]. In this DCE study, the authors find that the public place extremely low weights on the lifetime health of individuals in comparison to the magnitude of the health gain offered, and that these weights are marginally counter to the idea of aversion to inequalities in lifetime health. For example, the authors find the public place an incremental weight of 0.94 on an incremental health gain to someone with an age of death of 60, compared to someone with an age of death of 80.

## Discussion

This review set out to do three things. First, to identify estimates of the strength of the UK public’s aversion to inequalities in lifetime health between socioeconomic groups. Second, to explore whether the strength of this aversion differs depending upon the type of health under consideration. Third, to explore whether or not aversion differs depending upon whether participants were told that the inequality existed between socioeconomic groups, or neutrally framed groups. We identified 15 studies relevant to these aims.

The identified studies provide general, although not universal, support for the idea that the UK public are averse to inequalities in life expectancy (at birth) between socioeconomic groups. Similarly, the studies identified provide evidence that the public are averse to inequalities in life-expectancy (at birth) between neutrally framed groups in a comparable range of lifetime health. Eleven of the 15 studies identified provide evidence in support of aversion to inequalities in total life expectancy [6–9, 23, 24, 31, 33, 35, 38, 41], two provide evidence in opposition [36, 39], and two are inconclusive [37, 40]. However, the strength of aversion differed substantially between studies, with higher levels of aversion elicited for inequalities presented as being between socioeconomic groups than between neutrally framed groups. For example, Petrou et al. [33] and Baker et al. [35] estimate relative weights of only 1.37 and 1.55, respectively, on an incremental health gain provided to someone who will die at 60, compared to someone who will die at 80. In contrast, Dolan and Tsuchiya [8] estimate weights of 6.8–9.95 for a marginal health gain provided to an individual of lower socioeconomic status with a life-expectancy of 73 compared to an individual of higher socioeconomic status with a life-expectancy of 78. Similarly, it is notable that the Atkinson inequality aversion parameters estimated by Edlin et al. [24] in a neutral context are substantially lower than those estimated by Robson et al. [6], Ali et al. [7], and Dolan and Tsuchiya [8] in a socioeconomic context; see Table 3.

A small number of the identified studies suggest that the public may be more averse to an inequality of a given QALY magnitude if that inequality is due to differences in life-expectancy, rather than quality of life. Both Rowen et al. [31] and Dolan and Tsuchiya [23] find that, whilst the public are averse to inequalities in quality-adjusted life expectancy (QALE) attributable to differences in life-expectancy, they are not averse [31], or as averse [23], to inequalities in QALE attributable to differences in quality of life. Similarly, in their profile tests, Baker et al. [35] find that the public prefer to prioritise those with better, rather than worse, past quality of life. This evidence suggests that public preferences regarding inequalities in health may be consistent with the “fair innings” argument based on duration of life, but may not be consistent with the “extended fair innings” argument that adjusts for quality of life [30]. None of the studies identified explored the possibility that health inequality aversion might depend upon the specific type of health gain under consideration (e.g., comparing aversion in the context of pain relief and life extension).

This review has three primary limitations. First, our inclusion of studies focused on BOI under the assumption that the impact of BOI on preferences is linear, which is an assumption that may or may not hold [31]. Sensitivity analysis indicates that the exclusion of the two BOI studies identified would not have an impact upon our conclusions regarding aversion to life-expectancy at birth. However, one of the three studies that suggests aversion to inequalities in lifetime health attributable to differences in quality of life may be lower than to those attributable to differences in length of life was a BOI-based study [31]. As a result, the strength of this conclusion would be weakened by excluding these studies. Second, our search was designed to inform distributionally sensitive economic evaluations conducted in the UK, and so was restricted to evidence on the views of people in the UK. As a consequence, the results themselves may be of limited generalisability to other countries. Third, the studies identified are methodologically heterogeneous, and report estimates of aversion in different ways. This makes it challenging to compare across studies and, with the exception of the four studies for which we calculated Atkinson inequality aversion parameters, it prevents any attempt at formal synthesis. The primary strength of this paper is the fact that it is the first systematic review of this kind; notably, we identified more studies than found in a recent unsystematic review of health inequality aversion [21].

### Four key issues

Our findings raise four issues. First, if the public are averse to inequalities in health, does it make sense to continue to conduct, and use, distributionally naïve economic evaluations? [43]. Whilst this review demonstrates that it is challenging to quantify precisely how averse the public are to inequalities in health, the evidence available does suggest they are averse. The distribution of health gains appears to matter to the UK public, and ignoring this preference by continuing to conduct distributionally naïve economic evaluation is a choice that runs counter to this preference. Second, if we want to introduce consideration of inequalities into economic evaluation, what level, or levels, of aversion should be implemented in practice?^19^ This is a critical question, because the prioritisation of equality has a human cost [30, 44]. If we choose to prioritise equality, we accept there will be more suffering, and loss of life, than might otherwise be present in our society. Conversely, if we choose not to prioritize equality, we choose to accept that the social burden of ill health will be disproportionately placed on the poor. The level of inequality aversion incorporated in an economic evaluation would quantify the acceptable human cost of a given improvement in equality, and so it is critical to define it in a considered way. This review found wide variation in estimates of public preferences regarding inequalities across studies, which highlights the challenge of selecting a single estimate of aversion to implement. Given this variation, those conducting economic evaluations would be wise to undertake sensitivity analyses surrounding the relative weight they give to the distribution of health gains and average population health gains. If distributionally sensitive economic evaluation is to become more widespread in the UK, it would be valuable for a body like NICE or Public Health England to define a reference level of health inequality aversion (perhaps using a Citizen’s Council comparable to [40]), so that those conducting these analyses can present their work in a comparable and consistent manner. Again, note that if these bodies do not comment on this issue, this equates to an endorsement of a status quo in which the reduction of inequalities in health carries no weight in economic evaluation. Third, if aversion to socioeconomic inequalities in health is higher than aversion to neutrally framed inequalities of equivalent magnitude, which (if either) strength of aversion is the appropriate one to reflect in distributionally sensitive economic evaluation? Should estimates of aversion from neutrally framed studies be used because this removes the influence of non-health factors upon respondents’ preferences? Or should estimates of aversion from socioeconomically framed studies be used because this reflects the fact that inequalities in health between socioeconomic groups are systematic, as opposed to being random variation within the population, and so may be considered inequitable? Fourth, is health inequality aversion consistent with the QALY model, or does the type of health matter to the public? If aversion does differ depending upon whether the public are asked about life-expectancy, pain relief, or any other form of health gain: how should this be accounted for in distributionally sensitive economic evaluation? Can QALY-based distributional cost-effectiveness analysis represent the views of the public?

In conclusion, this review suggests that the UK public are averse to inequalities in life expectancy between socioeconomic groups, albeit with wide variation in the strength of this preference between studies. We find evidence of aversion between neutrally framed groups; however, the UK public appears to be more averse to inequalities in health between socioeconomic groups. We find limited evidence that the composition of an inequality may impact the strength of aversion, and in particular, that the public may be less averse to an inequality of a given QALY magnitude if that inequality is due to differences in quality of life, rather than life-expectancy.

## Electronic supplementary material

Below is the link to the electronic supplementary material.

Supplementary material 1 (DOCX 17 kb)

## Footnotes

1 A finding mirrored in Spain [45, 46], the United States [47] and Australia [25].

2 Note that this keyword supplementation means our search strategy captures both the keywords anticipated by the authors, and those used in the “pearls” [22]. Also, note that an iterative procedure was used to ensure that the search picked up all papers referenced in any of the identified papers. The combination of these two factors means that it is unlikely that the choice of pearls will have artificially narrowed the number of studies identified; although a non-pearl based, non-iterative procedure may have missed some of the studies we identified.

3 This included samples recruited from limited geographical areas. These samples were included, as we were aware that practicality means that face-to-face research is commonly conducted in limited geographical areas. We did not want to bias our results in favour of online, geographically broad samples, and so opted to include these geographically limited studies.

4 For the avoidance of doubt, when we refer to “life expectancy” we refer to life expectancy from birth, and not remaining life-expectancy from a given point in time.

5 This includes studies focused upon the “fair innings” argument, and its “extended” version that incorporates quality of life [30]—the idea that those individuals who are not expected to experience a ‘normal’ span of lifetime health should be prioritised over those who are. Note that lifetime health is expressed in terms of life expectancy from birth in the fair Innings argument, and in terms of quality adjusted life expectancy from birth in the extended fair Innings argument.

6 Note that tests of BOI are founded upon shortfall in prospective health, rather than lifetime health. However, if we assume the impact of BOI on the preferences of the public is linear—as the literature on BOI does [31]—then estimates of preferences regarding BOI may be used to imply aversion to inequalities in lifetime health.

7 Note that one study featured both a UK level, and local sample [7].

8 Note that Lancsar et al. [36] and Baker et al. [35] are both part of the social value of a QALY project, and that [35] is an overarching report that contains the results of the discrete choice experiment reported in [36], and other parts of the project, including a person trade off (PTO) study. Throughout this paper, when we refer to the Baker et al. [35] study we are referring to this PTO study.

9 When we refer to “simple choice questions” we mean questions did not present concrete outcome scenarios, and were more generally about prioritisation. In contrast, the “other forms” of exercise were more traditional choice experiments featuring outcomes, but that were not BTO/PTO studies.

10 Decomposed = A health profile expressed in terms of length of life, and quality of life, not expressed as a QALY value. Composed = A health profile expressed in terms of QALYs.

11 Class I = professional occupation, Class V = unskilled.

12 In these cases, the median respondent violated monotonicity and preferred not to grant an incremental health benefit to the better off group, even when it came at no opportunity cost to the worse off group.

13 Note that the weights presented for [6] and [7] are not directly comparable to those for [8], as [8] used a different baseline inequality than was tested in [6] and [7]. If the Atkinson inequality aversion parameter from [8] is applied to the inequality tested in [6] and [7], this produces an implied weight of 166.22 on a marginal gain to a poorer individual compared to a richer individual [(74/62)^(27.9 + 1)]—see [8] for details of how these weights are derived.

14 If the Atkinson inequality aversion parameters (ɛ) estimated by Edlin are applied to the inequality tested in [6] and [7], this results in estimated weights on marginal gains to the group with lower health of 3.86 and 2.77 for A [(74/62)^(6.63 + 1)] and B, respectively, [(74/62)^(4.76 + 1)].

15 See Table 5 in Petrou et al. [33]: 0.78/0.57 = 1.37.

16 PQ2, Q1, Q2 and Q3.

17 Note that the questions themselves featured both form of BOI simultaneously, and that this effect is a product of the way these two were decomposed in the analysis undertaken.

18 See Table 20 in [35]: 0.814/0.527 = 1.55.

19 Note that this point applies equally to the development of indices of population health that account for aversion to inequalities in health [48, 49].
