# Supplementary material for: How averse are the UK general public to inequalities in health between socioeconomic groups? A systematic review
Source: Eur J Health Econ. 2019 Oct 24;21(2):275–85. doi: 10.1007/s10198-019-01126-2 (PMC7072057; doi:10.1007/s10198-019-01126-2)
Supplement: Supplementary file 1 — Supplementary material 1 (DOCX 17 kb) [file 10198_2019_1126_MOESM1_ESM.docx]

Supplementary Online Appendix 1

Figure 1. MEDLINE Search Strategy

1. Choice Behaviour/ or Quality-Adjusted Life Years/ or Health Priorities/ or Health Care Rationing/ or Value of Life/
2. (Health or QALY or Quality Adjusted Life Year*).ti,ab.
3. 1 or 2
4. (respondent* or sampl* or participant* or subjects of electoral register or electoral roll).ti,ab.
5. (study or studies or survey* or experiment* or elicit* or empirical* or DCE or person trade-off or PTO or preference*).ti,ab.
6. 4 or 5
7. 3 and 6
8. ((distribution* adj weight*) or equity weight* or (QALY adj2 weight*) or (equity adj2 preference*) or (QALY and relative value)).ti,ab.
9. ((health adj maximi*) or health benefit maximi*).ti,ab.
10. (social value* or societal value*).ti,ab.
11. ((distribut* adj2 preference*) or distribut* criteria).ti,ab.
12. (outcome egalitaria* or gain egalitaria* or prioritaria* or sufficientaria*).ti,ab.
13. (public preference* or community preference* or societal preference*).ti,ab.
14. (rationing guideline* or prioritisation criteria or prioritization criteria).ti,ab.
15. (Social Welfare Function* or SWF).ti,ab.
16. Health Inequality Aversion.ti,ab.
17. (fair innings or egalitarian ageism or age-related weights or age-weighting preferences of (age and priority setting)).ti,ab.
18. (Absolute Shortfall or Proportional Shortfall).ti,ab.
19. (Burden of illness and (QALY* or Quality Adjusted Life Year*)).ti,ab.
20. 8 or 9 or 10 or 11 or 12 or 13 or 14 or 15 or 16 or 17 or 18 or 19
21. 7 and 20
